# Comparison of Achilles Tendon Loading Between Male and Female Recreational Runners

**DOI:** 10.2478/hukin-2014-0121

**Published:** 2014-12-30

**Authors:** Greenhalgh Andrew, Sinclair Jonathan

**Affiliations:** 1 London Sport Institute, Middlesex University.; 2 Division of Sport Exercise and Nutritional Sciences, University of Central Lancashire.

**Keywords:** biomechanics, gender, injuries, ankle

## Abstract

Recreational running is an activity with multiple reported health benefits for both sexes, however, chronic injuries caused by excessive and/or repetitive loading of the Achilles tendon are common. Males have been identified as being at an increased risk of suffering an injury to the Achilles tendon and as such, knowledge of differences in loading between the sexes may provide further information to better understand why this is the case. The aim of the current investigation was to determine whether gender differences in the Achilles tendon load exist in recreational runners. Fifteen male (age 26.74 ± 5.52 years, body height 1.80 ± 0.11 m and body mass 74.22 ± 7.27 kg) and fifteen female (age 25.13 ± 6.39 years, body height 1.68 ± 0.12 m and body mass 67.12 ± 9.11 kg) recreational runners volunteered to take part in the current investigation. Participants completed 10 trials running at 4.0 m·s^−1^ ±5% striking a force platform (1000 Hz) with their right foot. Ankle joint kinematics were synchronously recorded (250 Hz) using an optoelectric motion capture system. Ankle joint kinetics were computed using Newton-Euler inverse-dynamics. Net external ankle joint moments were then calculated. To estimate Achilles tendon kinetics the plantarflexion moment calculated was divided by an estimated Achilles tendon moment arm of 0.05 m. Differences in Achilles tendon kinetics were examined using independent sample t-tests (p<0.05). The results indicate that males were associated with significantly (p<0.05) greater Achilles tendon loads than females. The findings from this study support the notion that male recreational runners may be at greater risk of Achilles tendon pathology.

## Introduction

Recreational running is an activity with multiple reported health benefits for both sexes (Egan et al., 2006; Williams, 2012; Williams, 1997). Excessive repetitive loading of the Achilles tendon can lead to overuse injuries (Hess, 2010). Overuse injuries to the Achilles tendon can range from discomfort to an inability to run and have been reported as accounting for 8% and 10% of running related injuries in males and females, respectively (Taunton et al., 2003).

Research suggests a genetic predisposition to increased risk of Achilles tendon injury (Katz and Mubarak, 1984; Kraemer et al., 2012; Ribbans and Collins, 2013). Between the sexes, males have been identified as most at risk of Achilles tendon injury by a factor of 2:1 to 12:1 to their female counterparts (Hess, 2010; Houshian et al., 1998; Soma and Mandelbaum, 1994; Vosseller et al., 2013). In part this discrepancy between genders may be due to differences in the level of participation in ball sports which has been linked to high rates of Achilles injuries (Leppilahti et al., 1996).

It should be noted that during running, differences in kinetics and kinematics between sexes have been well established (Chumanov et al., 2008; Ferber et al., 2003; Nigg et al., 2012; Sakaguchi et al., 2012; Sinclair et al., 2012). However with specific reference to the ankle joint kinematics in the sagittal plane which can be used to calculate Achilles tendon loading (Scott and Winter, 1990), previous research identified no significant differences between sexes while running (McKean et al., 2007; Sinclair et al., 2012).

A reduction in intrinsic risk factors for Achilles tendon injury can be minimised through regular physical activity (Hess, 2010; Malvankar and Khan, 2011). While the benefits of physical exercise for tendon health are clear, a lack of rest between running sessions can lead to overall collagen degradation (Almonroeder et al., 2013). Achilles tendon injuries have been linked to longer duration of sports participation in terms of years as well as chronological age (Knobloch et al., 2008; Taunton et al., 2002; Waugh et al., 2012). It would appear that a correct balance of loading magnitude and frequency of the tendon interspersed with periods of rest may reduce the overall risk of an Achilles injury.

Understanding the gender differences in Achilles tendon loading during running may therefore provide insight into the aetiology of Achilles tendon pathology in runners and allow gender specific exercise recommendations to be made.

Despite the differences in injury rates of the Achilles tendon in males and females, there is a paucity of research investigating any potential differences in loading of the tendon during running between sexes. The aim of this research was to investigate if differences existed between the sexes in the loading of the Achilles tendon when normalised to bodyweight (BW), measuring tendon force during running. It was hypothesised that due to similar kinematics of the ankle joint no significant differences in Achilles tendon loading between the sexes would be observed.

## Material and Methods

### Participants

Fifteen male (age 26.74 ± 5.52 years, body height 1.80 ± 0.11 m and body mass 74.22 ± 7.27 kg) and fifteen female (age 25.13 ± 6.39 years, body height 1.68 ± 0.12 m and body mass 67.12 ± 9.11 kg) recreational runners volunteered to take part in the current investigation. All were free from lower extremity injury at the time of data collection and provided written informed consent. Ethical approval was obtained from the University of Central Lancashire School of Psychology.

### Procedures

Participants completed 10 trials running at 4.0 m·s^−1^ ±5% striking a force platform (Kistler, Kistler Instruments Ltd, Alton, Hampshire) (1000 Hz) with their right foot. The experimental laboratory was 22 m long. Running velocity was controlled using infra-red timing gates (SmartSpeed Ltd UK) spaced 4 m apart. The stance phase of the running cycle was identified as being the duration over which ≥20 N of vertical force was applied to the force platform (Sinclair et al., 2011). All participants wore Saucony Pro Grid Guide 2 running shoes to avoid any influence of footwear on Achilles tendon loading (Rowson et al., 2010). Kinematics were synchronously recorded (250Hz) by an 8 camera opto-electric motion capture system (Qualisys Medical, Gothenburg, Sweden).

In order to define the anatomical frames of the right foot and shank segments, this study used the calibrated anatomical systems technique (CAST) (Cappozzo et al., 1995). Retro-reflective markers were attached unilaterally to the calcaneus, 1st and 5th metatarsal heads, medial/lateral malleoli and medial/lateral femoral epicondyles. A rigid technical cluster comprised of four retroreflective markers mounted to a thin sheath of lightweight carbon fibre was securely attached to the anterior-lateral aspect of the shank segment. The foot segment was tracked using the metatarsal and calcaneus markers. Prior to data collection, a static trial was obtained which allowed the anatomical markers to be referenced in relation to the technical marker positions.

### Measures

Three-dimensional kinematics were smoothed using a low-pass Butterworth 4^th^ order zero-lag filter at a cut off frequency of 12 Hz and were then quantified using Visual 3-D (C-Motion Inc, Germantown, USA). Three-dimensional rotations of the ankle joint were calculated using an XYZ cardan sequence of rotations (where X = sagittal plane; Y = coronal plane and Z = transverse plane rotation) (Sinclair et al., 2012).

Ankle joint kinetics were computed using Newton-Euler inverse-dynamics. Net external ankle joint moments were then calculated. Achilles tendon load (ATL) (B.W) was determined using a predictive technique by dividing the plantarflexion moment (PFM) by the estimated Achilles tendon moment arm (atma). This technique has been utilized previously to resolve differences in Achilles tendon load during running (Kulmala et al., 2013; Sinclair et al., 2014). The moment arm was quantified as a function of the ankle sagittal plane angle (*AKA*) using the procedure described by Self and Paine (2001):

ATL=PFM/atma

$$\text{atma}=-0.5910+0.08297\;\mathrm{AKA}-0.0002606\;\mathrm{AK}A^{2}$$

Average and instantaneous ATL loading rates (B·W·s^−1^) were calculated in accordance with the procedure outline by Sinclair et al. (2014).

### Statistical analyses

Descriptive statistics (Means ± SD) were calculated for both male and female runners. Differences in Achilles tendon kinetics were examined using independent sample t-tests with significance accepted at the level of p≤0.05 (Rothman 1990). Statistical procedures were conducted using SPSS version 20.0 (SPSS Inc, Chicago, USA).

## Results

Table 1 presents the Achilles tendon loading parameters observed from the current study as a function of sex.

### Achilles tendon force

The results show that males were associated with significantly greater peak PFM compared to females t (28) = 2.65, p<0.05, D = 1.00. Males also exhibited a significantly greater ATL t (28) = 2.11, p<0.05, D = 0.80 compared to females. Finally both average t (28) = 2.21, p<0.05, D = 0.84 and instantaneous t (28) = 2.36, p<0.05, D = 0.89 ATL loading rates were shown to be significantly greater in male runners.

## Discussion

The aim of this research was to investigate whether gender differences in the Achilles tendon load were present in recreational runners. This study represents the first comparative investigation to examine the differences in Achilles tendon loading during running and may provide insight into an increased incidence of Achilles tendon pathologies in males.

In support of the hypothesis the key observation from the current investigation is that Achilles tendon load parameters were significantly greater in male runners in comparison to females. This finding has potential clinical relevance with regard to the aetiology of Achilles tendon pathology in recreational runners. The pathogenesis of Achilles tendon injury is considered to be mediated through excessive and habitual loading of the tendon during dynamic movements such as running (Magnusson et al., 2010). When the magnitude and frequency of the load experienced by the tendon during running falls outside the levels that are tolerable by the tendon itself this results in degeneration of the tendon (Selvanetti et al., 1997).

Sufficient rest between bouts of running has been shown to mediate a physiologically positive influence on the tendon. This occurs through collagen synthesis, that produces hypertrophic alterations in the tendon itself (Magnusson et al., 2010). When insufficient rest is allowed this means that tendon degradation outpaces synthesis and the tendon becomes weakened, thus, its susceptibility to injury increases (Magnusson et al., 2010). The current investigation may therefore provide insight into the mechanism by which males are associated with an increased incidence of Achilles tendon pathology compared to females.

In conclusion, the observations of the current investigation show that male recreational runners are associated with greater Achilles tendon load parameters in comparison to females. Given the proposed relationship between tendon loading and the aetiology of tendon pathology, the current investigation provides further insight into an increased incidence of Achilles tendon pathologies in males. Future research should seek to investigate other mechanisms linked to the aetiology of Achilles tendon pathologies and also the influence of different intervention strategies aimed at reducing the load experienced by the Achilles tendon in male runners.

## Figures and Tables

**Table 1:** Achilles tendon force measurements as a function of gender

	**Male**	**SD**	**Female**	**SD**

Peak force (BW)	5.22	0.86	4.94	0.45
Time to peak force (ms)	132.10	27.90	133.91	16.69
Average tendon loading rate (BW·s^−1^)	42.60	16.11	37.70	5.81
Instantaneous tendon loading rate (BW·s^−1^)	121.31	49.92	110.04	18.96
